# Evaluation of Safety and Protective Effect of Combined Extract of *Cissampelos pareira* and *Anethum graveolens* (PM52) against Age-Related Cognitive Impairment

**DOI:** 10.1155/2012/674101

**Published:** 2012-09-18

**Authors:** Wipawee Thukham-mee, Jintanaporn Wattanathorn

**Affiliations:** ^1^Neuroscience Program Department of Physiology and Graduate School, Faculty of Medicine, Khon Kaen University, Khon Kaen 40002, Thailand; ^2^Department of Physiology, Faculty of Medicine, Khon Kaen University, Khon Kaen 40002, Thailand; ^3^Integrative Complementary Alternative Medicine Research and Development Group, Khon Kaen University, Khon Kaen 40002, Thailand

## Abstract

The present study aimed to determine acute toxicity, the protective effect, and underlying mechanism of PM52, a combined extract of *Cissampelos pareira* and *Anethum graveolens,* against age-related cognitive impairment in animal model of age-related cognitive impairment. PM52 was determined as acute toxicity according to OECD guideline. Male Wistar rats, weighing 180–220 g, were orally given PM52 at doses of 2, 10, and 50 mg/kg at a period of 14 days before and 7 days after the bilateral administration of AF64A via intracerebroventricular route. All animals were assessed according to spatial memory, neuron density, MDA level, the activities of SOD, CAT, GSH-Px, and AChEI effect in hippocampus. It was found that all doses of PM52 could attenuate memory impairment and neurodegeneration in hippocampus. The possible mechanisms might occur via the suppression of AChE and the decreased oxidative stress in hippocampus. Therefore, our data suggest that PM52 may serve as food supplement to protect against age-related cognitive impairment such as mild cognitive impairment (MCI) and early phase of Alzheimer's disease. However, further researches are still essential.

## 1. Introduction

Aging is a phenomenon leading to the dysfunction of normal cellular regulation including cognitive function. As the age advances, the cognitive capability is declined. Since the cognitive decline is the most costly, in terms of the financial, personal, and societal, it is regarded as a major health and social issue burden. Therefore, age-related cognitive memory impairment is one of the important health problems that should be concerned.

Current studies demonstrate that cognitive impairment in both aged human and rodents is correlated with the accumulation of oxidative damage to lipids, proteins, nucleic acids [1–3], and the vulnerability of various neurotransmitters to oxidative stress [4–6]. Moreover, it is also reported to be related to the hypofunction of cholinergic system and manifested by the decreased acetylcholinesterase (AChE), one of the major markers of cholinergic function in various cerebral areas [7]. Since the present strategy has not yet been met by effective symptomatic treatments or preventative strategies, the novel strategy to protect against age-related cognitive decline is still required. Based on the role of oxidative stress and cholinergic system mentioned earlier, it has provided the rationale for protecting and treating age-related cognitive decline with substances targeting at enhancing cholinergic function and decreasing oxidative stress.

In traditional practices of medicine, numerous plants have been used to enhance cognitive function both in healthy individuals and those with diseases states such as mild cognitive impairment (MCI) and Alzheimer's disease (AD). The herbal medicine can be used either by single herb or by polyherbal formulation. The concept of polyherbalism is very peculiar to Oriental Medicine such as Ayurveda and Traditional Chinese Medicine (TCM). It is believed that the polyherbalism can provide high efficiency partly due to synergism. Therefore, the herbal ingredients in these formulations are selected based on their healing property with respect to the disease condition such as antioxidant and acetylcholine inhibitory (AChEI) effects. In order to assure the safety and therapeutic efficacy, this study aimed to determine the acute toxicity and cognitive-enhancing effect of combined extract of *Cissampelos pareira* and *Anethum graveolens* or PM52. Moreover, the possible underlying mechanism was also further explored.

## 2. Materials and Methods

### 2.1. Plant Materials and Preparation of Combined Extract

All plants materials used for the preparation of extract were purchased from organic farms of Srithat District, Udon Thani province. They were identified morphologically, histologically and authenticated by Associate Professor Panee Sirisa-ard, Faculty of Pharmacy, Chiang Mai University. Voucher specimens were kept at Integrated Complimentary Alternative Medicine Research and Development Group, Khon Kaen University. Powders of the *Cissampelos pareira* and *Anethum graveolens* were mixed at a ratio of 1 : 5 and extracted with hydroalcoholic solvent at the concentration of 50 : 50. The yielded extract was freeze-dried to powder with evaporator.

### 2.2. Determination of Total Phenolic Compounds

Total phenolic compounds of combined extract of *Cissampelos pareira* and *Anethum graveolens* were determined total phenolic compounds using Folin-Ciocalteau method. In brief, an aliquot of 0.1 mL of plant extract was added to 1.9 mL of deionized water and 1.0 mL of Folin-Ciocalteu phenol reagent (Sigma). After 8 min, 5.0 mL of 20% Na_2_CO_3_ was added and the mixture was heated in a boiling water bath for 1 min comparatively to gallic acid standard. Absorbance was measured at 765 nm with a UV-spectrophotomet (Pharmacia LKB-Biochrom4060) after cooling in darkness and the result was expressed as mg Gallic acid equivalents (GAE)/100 g extract.

### 2.3. Determination of Total Flavonoid

Total flavonoid content in extract of *Cissampelos pareira* and *Anethum graveolens* was determined via colorimetric method. Briefly, 0.5 mL of each sample and 300 *μ*L of NaNO_2_ (1 : 20 w/v) were pipette into a test tube. The contents were vortexed for 10 s and left at room temperature for 5 min. The mixture was then added 300 *μ*L of AlCl_3_ (1 : 10 w/v), 2 mL of 1 M NaOH, and 1.9 mL of distilled water. After 10 s of vortexing, the absorbance for each sample was measured at 510 nm. Quercetin was used as reference compound to produce standard curve and the result was expressed as g of quercetin equivalents (QE)/g of extract.

### 2.4. Animals

Healthy male Wistar rats (180–220 grams, 8 weeks old) were obtained from National Laboratory Animal Center, Salaya, Nakhon Pathom. They were housed in group of 4 per cage in standard metal cages at 22 ± 2°C on 12 : 12 h light-dark cycle. All animals were given access to food and water ad libitum. The experiments were performed to minimize animal suffering in accordance with the internationally accepted principles for laboratory use and care of European Community (EEC directive of 1986; 86/609/EEC). The experimental protocols were approved by the Institutional Animal Care and Use Committee (AE006/54).

### 2.5. Experimental Protocol

All rats were randomly assigned to 7 groups of 8 animals each.

Group I Vehicle + ACSF: rats were treated with vehicle at a period of 14 days before and 7 days after the administration of artificial cerebrospinal fluid (ACSF) via intracerebroventricular route bilaterally.

Group II Vehicle + AF64A: rats had been treated with vehicle for 14 days before and 7 days after the administration of AF64A, a cholinotoxin, in order to induce a cholinergic deficit as observed in MCI and early phase of AD.

Group III Donepezil + AF64A: animals were treated with Donepezil, a cholinesterase inhibitor which used as standard treatment for cognitive impairment. This group was used as positive control in this study.

Group IV Vitamin C + AF64A: animals were treated with Vitamin C (250 mg/kg BW), a well-known antioxidant which previously showed the neuroprotective and cognitive-enhancing effects. This group was also used as positive control in this study.

Group V–VII combined extract of *Cissampelos pareira* and *Anethum graveolens* + AF64A: rats had been treated with the polyherbal extract at various doses ranging from 2, 10, and 50 mg/kg BW for 14 days before and 7 days after the administration of AF64A, respectively, (the doses used in this study were selected based on our preliminary data on the cognitive-enhancing effect).

The animals determined the spatial memory at 7 days after AF64A administration.

Then, they were sacrificed and determined the density of survival neurons and in various subregions of hippocampus.

### 2.6. AF64A Administration

AF64A was prepared as an aqueous solution of acetylethylcholine mustard HCl (Sigma, St. Louis, MO, USA) and was adjusted to pH 11.3 with NaOH. After stirring for 30 min at room temperature, the pH was lowered to 7.4 with the gradual addition of HCl and stirred for 60 min. The amount of AF64A was then adjusted to 2 nmol/2 *μ*L. The vehicle of AF64A was distilled water prepared in the same manner as the AF64A and recognized as ACSF. In order to administer AF64A bilaterally via intracerebroventricular (i.c.v.) route, the animals were anesthetized with the intraperitoneal injection of sodium pentobarbital at dose of 60 mg/kg BW. Then, AF64A (2 nmol/2 *μ*L) was infused bilaterally via intracerebroventricular (i.c.v.) route with a 30-gauge needle inserted through a burr hole drilled into the skull into both the right and left lateral ventricles. Stereotaxic coordinates were (from the bregma) posterior 0.8 mm, lateral ±1.5 mm, and ventral (from dura) 3.6 mm. The rate of infusion was 1.0 *μ*L/min and the needle was left in place for 5 min after infusion and then slowly withdrawn.

### 2.7. Morris Water Maze Test

The Morris water maze test is one of the most important paradigms used for testing spatial navigation task, which is thought to be dependent on the proper functioning of the hippocampus. The testing apparatus for all tasks used in this study was a stainless-steel circular pool that 147 cm in diameter and 47 cm in depth. The interior of the pool was flat and the pool was placed on the steady floor. The pool was filled with water to a depth of 12 cm. The water was maintained at 23 ± 1°C and darkened by nontoxic powder.

The pool was divided into four quadrants (NE, NW, SE, and SW) by two imaginary lines crossing the center of the pool. For each animal, the invisible platform was placed in the center of one of the quadrants and remained there for a training period of 4 days. Each rat was gently placed in the water facing the wall of the pool from one of the four starting points (N, E, S, or W) along the perimeter of the pool, and the animal was allowed to swim until it climbed onto the platform. When an animal could not reach the platform in 60 s, it was gently placed on the platform by the experimenter. In either cases, the animal was left on the platform for 10 s and removed from the pool. Then, it was quickly dried with a towel before being returned to the home cage. The time which animal spent to find the immersed platform was regarded as escape latency. The 24 hr after the determination of escape latency, rats were reexposed to the same condition except that the immersed platform was removed and the time which the animal spent in the quadrant previously located in the immersed platform was recorded as retention time.

### 2.8. Histological Procedure

Following anesthesia with sodium pentobarbital (60 mg/kg BW), fixation of the brain

was carried out by transcardial perfusion with fixative solution containing 4% paraformaldehyde in 0.1 M phosphate buffer pH 7.3. The brains were removed after perfusion and stored over a night in a fixative solution that used for perfusion. Then they were infiltrated with 30% sucrose solution for approximately 4°C. The specimens were frozen rapidly and 30 *μ*M thick sections were cut on cryostat. They were rinsed in the phosphate buffer and picked up on slides coated with 0.01% of aqueous solution of a high molecular weight poly L-lysine.

### 2.9. Morphological Analysis

Five coronal sections of each rat in each group were studied quantitatively. Neuronal counts in hippocampus were performed by eye using a 40x magnification with final field 255 *μ*m^2^ according to the following stereotaxic coordinates: AP −4.8 mm, lateral ±2.4–6 mm, and depth 3–8 mm. The observer was blind to the treatment at the time of analysis. Viable-stained neurons were identified on the basis of a stained soma with at least two visible processes. Counts were made in five adjacent fields and the mean number extrapolated to give total number of neurons per 255 *μ*m^2^. All data are represented as number of neurons per 255 *μ*m^2^.

### 2.10. Determination of Malondialdehyde Level and Acetylcholinesterase Activity

Hippocampus was isolated and prepared as hippocampal homogenate and the determination of the malondialdehyde (MDA) level and acetylcholinesterase (AChE) activity in hippocampus was performed. Malondialdehyde was indirectly estimated by determining the accumulation of thiobarbituric acid reactive substances (TBARS) while the activity of AChE was determined using Ellman method.

### 2.11. Determination of Scavenging Enzymes Activities

In order to determine the activities of antioxidant enzymes including superoxide dismutase (SOD), catalase (CAT), and glutathione peroxidase (GSH-Px), the brain tissues were weighed and homogenized with a buffer consisting of 10 mM sucrose, 10 mM Tris-HCl, and 0.1 mM EDTA (pH 7.4). Then the brain homogenates were centrifuged at 3000 g for 15 min at 4°C. The supernatant was used for bioassays. The activity of SOD was determined using a xanthine/xanthine oxidase system for the production of superoxide radical and subsequent measurement of cytochrome *c* as a scavenger of the radicals. Optical density was determined using a spectrometer (UV-1601, Shimadzu) at 550 nm. One unit of enzyme activity was defined as the quantity of SOD required to inhibit the rate of reduction of cytochrome *c* by 50%. SOD activity was presented as units per milligram of protein (U mg^−1^ protein). CAT activity in the supernatant was measured by recording the rate of decrease in H_2_O_2_ absorbance at 240 nm. The activity of CAT was expressed as *μ*mol H_2_O_2_/min/mg protein. GSH-Px was determined using *t*-butylhydroperoxide as a substrate. The optical density was spectrophotometrically recorded at 340 nm. One unit of the enzyme was defined as micromole (*μ*mol) of reduced nicotinamide adenine dinucleotide phosphate (NADPH) oxidized per minute. GSH-Px activity was expressed as U/mg protein.

### 2.12. Statistical Analysis

Data are presented as mean ± standard error of mean (S.E.M). One-way analysis of variance (ANOVA), followed by Tukey post hoc test. A probability level less than 0.05 was accepted as significance.

## 3. Results

### 3.1. Total Phenolic Compounds and Flavonoids Contents in PM52

To date, the use of plant-based formulations is leading to a fast growing market for Ayurvedic, nutraceutical, and polyherbal formulations. The development of polyherbal formulation has been regarded as a challenging task because of the large number of varied chemical compounds present in the different medicinal plants can possibly provide more benefit. However, during the formulation of new drugs or the reformulation of existing products, the interaction between active markers of various plant extracts also occurs resulting in changes in the chemical nature and therapeutic response. Therefore, the characteristic of PM52 has been developed and determined in this study. It was found that the extract contained the total phenolic compounds at concentration of 582.09 ± 8.72 mg of GAE/100 g of plant extract and contained total flavonoids at concentration of 18.80 ± 0.25 mg of QE/100 g of plant extract. Therefore, a combined extract of *Cissampelos pareira* and *Anethum graveolens* contained more phenolic compounds and flavonoids than *Cissampelos pareira* or *Anethum graveolens* as shown in Table 1.

### 3.2. Acute Toxicity of PM52

In the acute toxicity study, it was found that PM52 up to the level of 5000 mg/kg BW failed to exhibit the lethality and toxic symptoms. No behavioral change and macroscopic changes of histology of vital organs were observed. Further dosing to evaluate the LD_50_ of PM52 had not been performed. According to the Organization of Economic Cooperation and Development (OECD) guidelines for acute oral toxicity, an LD_50_ of 2000 mg/kg BW or above is categorized as unclassified and hence the product is found to be safe. Therefore, PM52 is safe especially for short duration application.

### 3.3. Cognitive-Enhancing Effect and Neuroprotective Effect of PM52

**Figure 1:**
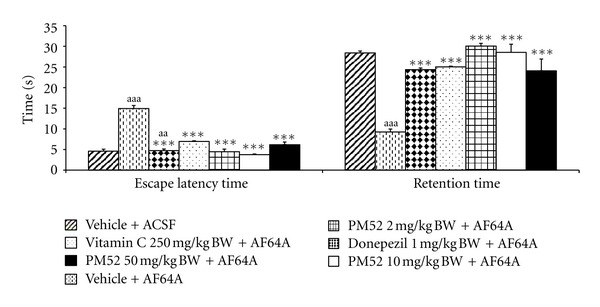
Effect of Donepezil, Vitamin C, and various doses of PM52 ranging from 2, 10, and 50 mg/kg BW on the escape latency and retention time evaluated using Morris water maze test in memory impairment rats induced by AF64A. Values are expressed as mean ± SEM. (*n* = 8/group) ***P value < .001 compared with vehicle plus ACSF-treated group, ***P value < .001 compared with vehicle plus AF64A-treated group.

Figure 1 showed the cognitive-enhancing effect of PM52 at doses of 2, 10, and 50 mg/kg BW on spatial memory in memory deficit rats induced by AF64A. Our data showed that Vehicle + ACSF showed no significant changes of both escape latency and retention time. These findings indicated that both vehicle which used to dissolved PM52 and ACSF which used to dissolved AF64A produced no effect on the mentioned parameters. Rats which exposed to AF64A significantly enhanced escape latency but decreased retention time (*P*-value < .001 all; compared to vehicle + ACSF). However, these changes could be mitigated by Donepezil, Vitamin C, and PM52 at all doses used in this study (*P*-value < .001 all; compared to vehicle + AF64A).

**Figure 2:**
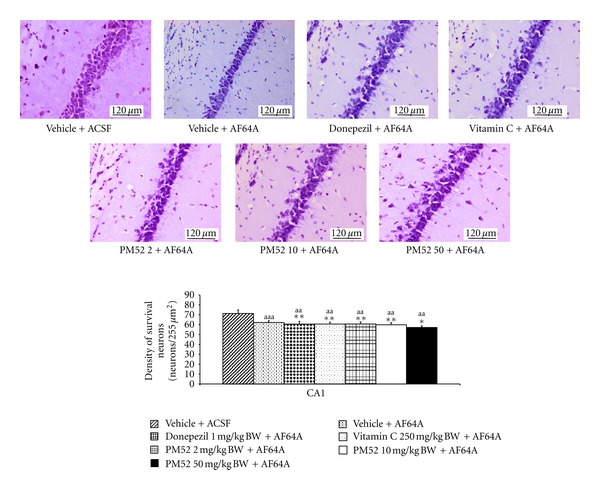
Effect of Donepezil, Vitamin C, and various doses of PM52 ranging from 2, 10, and 50 mg/kg BW on neuron density in CA1 of hippocampus of memory impairment rats induced by AF64A. Values are expressed as mean ± SEM. (*n* = 8/group) ^a,aa,aaa^ *P*-value < .05, .01 and .001 compared with vehicle plus ACSF-treated group, ^∗,∗∗,∗∗∗^ *P*-value < .05, .01 and .001 compared with vehicle plus AF64A-treated group.

**Figure 3:**
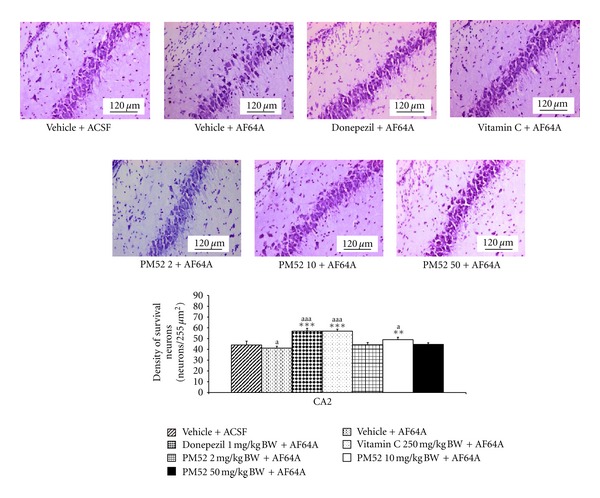
Effect of Donepezil, Vitamin C, and various doses of PM52 ranging from 2, 10, and 50 mg/kg BW on neuron density in CA2 of hippocampus of memory impairment rats induced by AF64A. Values are expressed as mean ± SEM. (*n* = 8/group) ^a,aa,aaa^ *P*-value < .05, .01 and .001 compared with vehicle plus ACSF-treated group, ^∗,∗∗,∗∗∗^ *P*-value < .05, .01 and .001 compared with vehicle plus AF64A-treated group.

**Figure 4:**
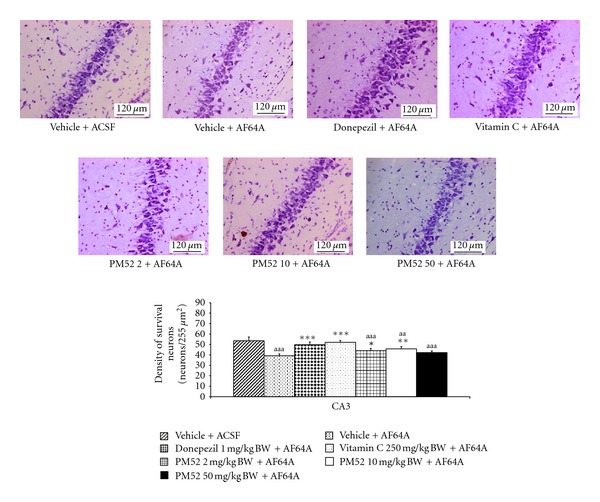
Effect of Donepezil, Vitamin C, and various doses of PM52 ranging from 2, 10, and 50 mg/kg BW on neuron density in CA3 of hippocampus of memory impairment rats induced by AF64A. Values are expressed as mean ± SEM. (*n* = 8/group) ^a,aa,aaa^ *P*-value < .05, .01 and .001 compared with vehicle plus ACSF-treated group, ^∗,∗∗,∗∗∗^ *P*-value < .05, .01 and .001 compared with vehicle plus AF64A-treated group.

**Figure 5:**
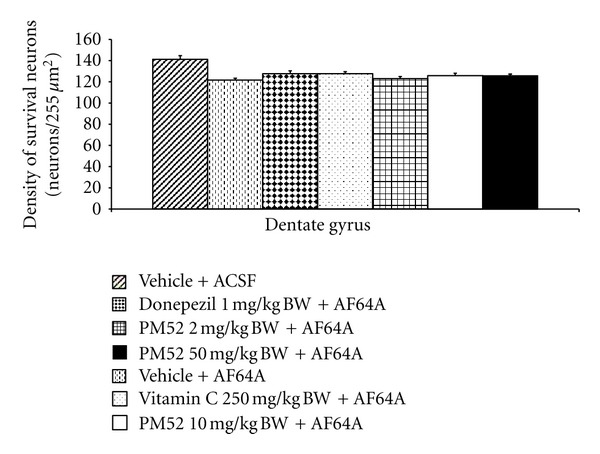
Effect of Donepezil, Vitamin C, and various doses of PM52 ranging from 2, 10, and 50 mg/kg BW on neuron density in dentate gyrus of hippocampus of memory impairment rats induced by AF64A. Values are expressed as mean ± SEM. (*n* = 8/group).

Since PM52 exerted the cognitive-enhancing effect, the effect of PM52 on the neuron density in hippocampus was investigated. The results were shown in Figures 2, 3, 4, and 5. The rats which received vehicle + AF64A showed the significant changes in CA1 (*P*-value < .001; compared to vehicle + ACSF) and CA3 (*P*-value < .001; compared to vehicle + ACSF). Rats treated with Donepezil or Vitamin C significantly mitigated the reduction of neuron density in CA1 (*P*-value < .01; compared to vehicle + AF64A) and CA3 (*P*-value < .001 all; compared to vehicle + AF64A). Interestingly, rats treated with either Donepezil or Vitamin C plus AF64A showed the increased neuron density in CA2 (*P*-value < .001 all; compared to vehicle + AF64A). It was found that treated with PM52 at doses of 2 mg/kg BW significantly increased neuron density in CA1 and CA3 (*P*-value < .01 and .05, resp., compared with vehicle + AF64A) while the medium dose treatment could enhance the neuron density in CA1, CA2, and CA3 (*P*-value < .01 all, compared with vehicle + AF64A). Moreover, the result showed that PM52 at doses of 50 mg/kg BW significantly increased neuron density in CA1 (*P*-value < .05, compared with vehicle + AF64A). No changes were observed in dentate gyrus.

### 3.4. Effect of PM52 on AChEI Activity and Oxidative Stress Markers

**Figure 6:**
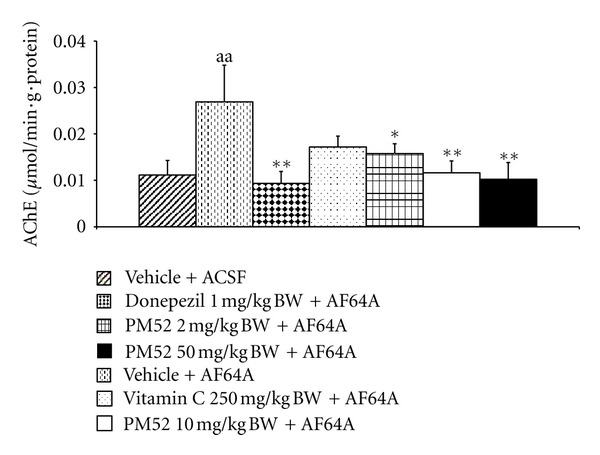
Effect of Donepezil, Vitamin C, and various doses of PM52 ranging from 2, 10, and 50 mg/kg BW on the acetylcholinesterase inhibitory activity in hippocampus of memory impairment rats induced by AF64A. Values are expressed as mean ± SEM. (*n* = 8/group) ^aa^ *P*-value < .01 compared with vehicle plus ACSF-treated group, **P-value < .01 compared with vehicle plus AF64A-treated group.

Since acetylcholine has been reported to play the crucial roles on cognitive function especially learning and memory, this study also focuses on the alteration of the mentioned transmitter and the activity of AChE was used to indicate the alteration of acetylcholine. The effect of PM52 on the activity of AChE in hippocampus was investigated and the results were shown in Figure 6. Rats which exposed to ACSF did not show a significant change of AChE activity whereas rats which exposed to AF64A showed the elevation of AChE (*P*-value < .01; compared to vehicle + ACSF). However, this change was reversed by Donepezil, Vitamin C, and PM52 at doses of 10 and 50 mg/kg BW (*P*-value < .01, .05, .01, and .01, resp.; compared to vehicle + AF64A).

The data obtained from previous part had revealed the neuroprotective effect of PM52. Based on the crucial role of oxidative stress on the pathophysiology of neurodegeneration, this part was focused on the effect of PM52 on oxidative stress markers including MDA level and the activities of scavenger enzymes including SOD, CAT, and GSH-Px.

**Figure 7:**
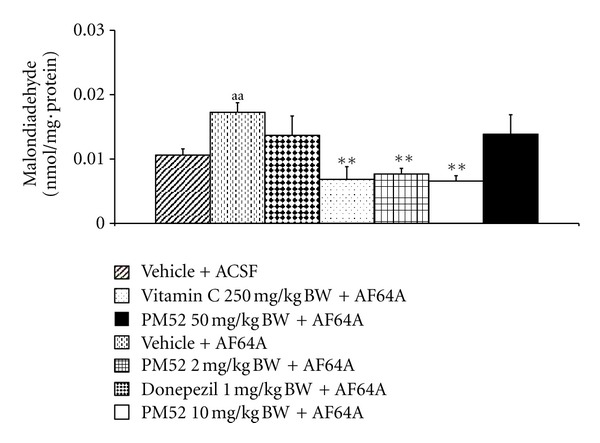
Effect of Donepezil, Vitamin C, and various doses of PM52 ranging from 2, 10, and 50 mg/kg BW on level of malondialdehyde (MDA) in hippocampus of memory impairment rats induced by AF64A. Values are expressed as mean ± SEM. (*n* = 8/group) ^aa^ *P*-value < .01 compared with vehicle plus ACSF-treated group, **P-value < .01 compared with vehicle plus AF64A-treated group.

Figure 7 showed that rats which exposed to ACSF did not produce any change on MDA level. Rats subjected to AF64A treatment revealed the decreased MDA level (*P*-value < .01; compared to vehicle + ACSF). This reduction was reversed by Vitamin C and PM52 both at dose of 2 and at dose of 10 mg/kg BW (*P*-value < .01 all; compared to vehicle + AF64A).

**Figure 8:**
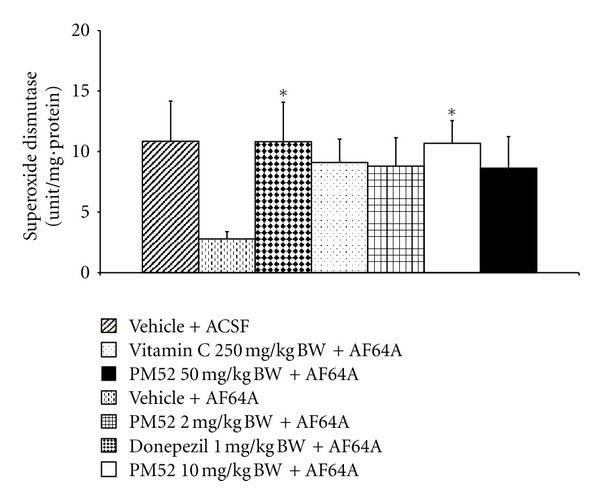
Effect of Donepezil, Vitamin C, and various doses of PM52 ranging from 2, 10, and 50 mg/kg BW on the superoxide dismutase (SOD) activity in hippocampus of memory impairment rats induced by AF64A. Values are expressed as mean ± SEM. (*n* = 8/group) *P-value < .05 compared with vehicle plus AF64A-treated group.

**Figure 9:**
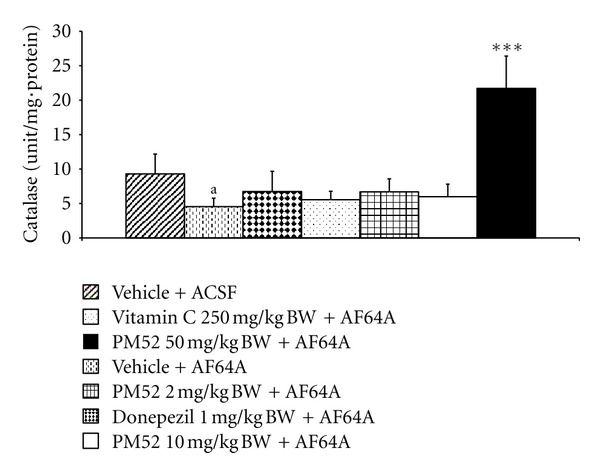
Effect of Donepezil, Vitamin C, and various doses of PM52 ranging from 2, 10, and 50 mg/kg BW on the catalase activity in hippocampus of memory impairment rats induced by AF64A. Values are expressed as mean ± SEM. (*n* = 8/group) ^a^ *P*-value < .05 compared with vehicle plus ACSF-treated group, ***P-value < .001 compared with vehicle plus AF64A-treated group.

**Figure 10:**
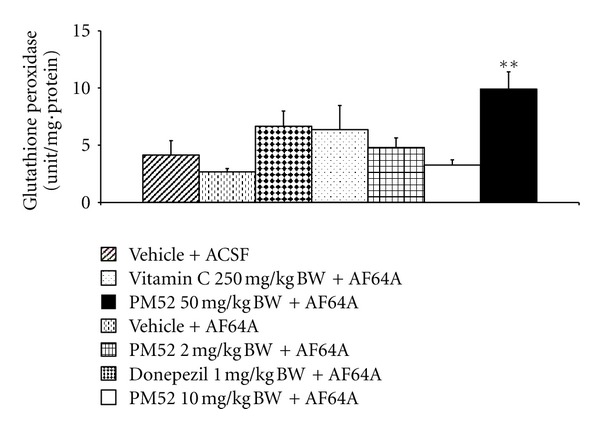
Effect of Donepezil, Vitamin C, and various doses of PM52 ranging from 2, 10, and 50 mg/kg BW on glutathione peroxidase (GSH-Px) activity in hippocampus of memory impairment rats induced by AF64A. Values are expressed as mean ± SEM. (*n* = 8/group) **P-value < .01 compared with vehicle plus AF64A-treated group.

The effect of PM52 on the activities of SOD, CAT, and GSH-Px activities was shown in Figures 8, 9, and 10. It was found that rats subjected to AF64A treatment reversed the decreased CAT and GSH-Px activities in hippocampus (*P*-value < .05 and .01 resp.; compared to vehicle + ACSF). The decreased CAT activity was reversed by the high dose of PM52 (*P*-value < .001; compared to vehicle + AF64A) while the decreased GSH-Px was reversed by Vitamin C and PM52 both at low and medium doses (*P*-value < .01 all; compared to vehicle + AF64A). In addition, rats which exposed to Donepezil and medium dose of PM52 and AF64A also enhanced the activity of SOD in the area just mentioned (*P*-value < .05 all; compared to vehicle + AF64A).

## 4. Discussion

Medicinal plants have long been used in traditional folklore in various cultures throughout the world. Recently, a scientific interest for phytotherapy has increased in various aspects especially the researches targeting at justifying the reputations of medicinal plants in traditional folklore and at their possible underlying mechanism.

PM52 showed not only cognitive-enhancing effect but also neuroprotective effect. PM52 could attenuate the neurodegeneration and could disturb the function of the affected areas. It has been reported that neurodegeneration occurs as the result of various factors including oxidative stress. Previous study demonstrated that oxidative stress is strongly scavenged by polyphenolic compounds including flavonoids which are found in herbal extracts [8]. Our data showed that PM52 significantly enhanced the activities of SOD, CAT, and GSH-Px in hippocampus and decreased MDA level in the mentioned area. The decrease in MDA level reflected the decreased attack of oxidative stress at lipid component, the main component of membrane including neuronal membrane resulting in the increased survival of neuron and cholinergic neuron in hippocampus. 

Hippocampus is regarded as a brain region essential for intact cognitive abilities and appears to be particularly vulnerable to the oxidative stress during aging [9, 10]. The neurodegeneration and the degeneration of cholinergic neuron in hippocampus contribute the important role on the spatial memory or hippocampal-dependent memory [11–15]. Therefore, the enhanced neuron density in hippocampus might also responsible in part for the cognitive-enhancing effect of PM52. 

**Figure 11:**
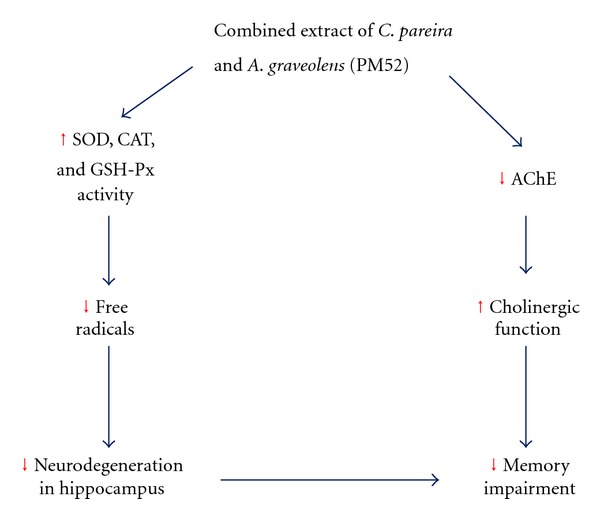
Schematic diagram of the possible underlying mechanism of PM52 to improve memory impairment in animal model of age-related cognitive decline condition such as mild cognitive impairment (MCI) and early phase of Alzheimer's disease.

Therefore, our data suggested that the cognitive-enhancing effect of PM52 might occur via 2 main mechanisms: (1) the suppression of AChE leading to the elevation of ACh, a neurotransmitter playing an important role on learning and memory and (2) the enhanced neuron density in hippocampus via the decreased oxidative stress induced by the increased antioxidant enzyme activities as shown in Figure 11. The possible active ingredient which contributes the role on the neuroprotective and cognitive-enhancing effect might be polyphenolic compounds especially quercetin which previously showed both cognitive-enhancing effect and neuroprotective effect [16, 17]. However, the influence of interaction effects of various ingredients still cannot be omitted.

In this study, PM52 failed to show dose-dependent manner in both cognitive-enhancing effect and neuroprotective effect. The possible explanation might be related to the nonsimple relationship between the concentration of PM52 and the interested parameters such as spatial memory and neuron density. Since both the memory and survival of neuron were under the influence of numerous factors, it was not possible to observe the simple relationship between the concentration of PM52 and the interested parameters. In addition, PM52 is the combined extract of *Cissampelos pareira* and *Anethum graveolens* and all ingredients are in the form of crude extract not a pure substance. Therefore, the effect of active ingredient was possibly masked by the other ingredients.

## 5. Conclusions

The results obtained from this study confirm that PM52, a polyherbal formulation of ethanolic leaves extracts of *Cissampelos pareira* and *Anethum graveolens*, provides the beneficial effect on the nervous system. It can enhance learning and memory in memory deficit condition especially in age-related cognitive decline such as mild cognitive impairment and early phase of Alzheimer's disease. In addition, PM52 also shows neuroprotective effect. The possible underlying mechanism occurs partly via the enhanced acetylcholine and the decreased oxidative stress. PM52 can provide beneficial effect at low dose while safety range is very wide. Therefore, PM52 can be served as adjuvant or complimentary therapy against age-related cognitive impairment. However, this study is only the preliminary study and further studies are necessary to fully elucidate the possible active ingredients, the detail mechanism of action, and subchronic toxicity of the polyherbal formulation. Moreover, further development of the standardized product of PM52 so that it can be used easily at home when required is still necessary.

## Figures and Tables

**Table 1:** Total phenolic compounds and total flavonoids content in *Anethum graveolens*, *Cissampelos pareira,* and PM52.

Tested substance	Total phenolic compounds	Total flavonoids
	(mg of GAE/100 g of plant extract)	(mg QE/g of plant extract)
*Anethum graveolens *	462.80 ± 4.59	10.90 ± 0.11
*Cissampelos pariera*	404.56 ± 3.06	8.38 ± 0.01
PM52	582.09 ± 8.72	18.80 ± 0.25
